# Post-COVID-19 increases in depression and other psychiatric disorders among Saudi children and adolescents

**DOI:** 10.3389/fpsyt.2026.1754791

**Published:** 2026-03-19

**Authors:** Ahmed M. Almai, Jay A. Salpekar, Anwar Alotaibi, Abdulsamad Al Jishi, Bayan Alabdulbaqi

**Affiliations:** 1 Psychiatry Department, Johns Hopkins Aramco Healthcare, Dhahran, Saudi Arabia; 2 Department of Psychiatry and Behavioral Sciences, Johns Hopkins University School of Medicine, Baltimore, MD, United States; 3 Research Office, Johns Hopkins Aramco Healthcare, Dhahran, Saudi Arabia

**Keywords:** anxiety, child psychiatry, COVID-19, depression, eating disorders, Saudi Arabia, sleep disorders

## Abstract

**Background:**

The COVID-19 pandemic produced unprecedented disruption in the daily lives of children and adolescents worldwide, increasing vulnerability to emotional and behavioural difficulties.

**Objective:**

To evaluate changes in psychiatric diagnostic patterns among pediatric referrals before and after the COVID-19 pandemic in a large Saudi tertiary healthcare system and compare these trends with Gulf and international findings.

**Methods:**

A retrospective cross-sectional review was conducted of new psychiatric referrals aged 3–18 years at Johns Hopkins Aramco Healthcare (JHAH), Dhahran. Pre-pandemic data included all referrals from 2019. Post-pandemic service-recovery data included referrals from January–November 2021. Routine psychiatric outpatient clinics were fully suspended during 2020 due to institutional COVID-19 emergency policy, meaning no routine outpatient psychiatric referrals occurred that year. To avoid bias associated with service suspension, 2020 was excluded from analysis. Diagnoses were grouped using ICD-10 categories. Descriptive and inferential statistics were applied.

**Results:**

Significant increases were observed in depressive disorders, anxiety disorders, sleep–wake disorders, feeding and eating disorders, and total psychiatric referrals after the pandemic. These findings closely parallel Gulf regional and international literature.

**Conclusion:**

The COVID-19 pandemic was associated with marked increases in psychiatric morbidity among Saudi youth. Expanded screening, early intervention and service capacity are required to mitigate long-term impact.

## Introduction

COVID-19 caused global disruption affecting children’s education, social development and psychological wellbeing (1–4). In Saudi Arabia, lockdown and routine disruption were associated with heightened psychological stress among children and adolescents (5). Similar findings were reported in the UAE and Qatar (6, 7), indicating shared regional vulnerability.

International research has demonstrated large increases in depressive and anxiety symptoms among youth, with pooled prevalence estimates of 25% and 20% respectively during the pandemic (8). Global burden modelling confirmed sharp increases in depressive and anxiety disorders associated with pandemic stressors (9). Emergency-department mental-health presentations in adolescents rose significantly, particularly among females (10, 11). Sleep disruption (12) and eating-disorder severity and admissions (13, 14) also increased across multiple countries.

Despite this emerging literature, service-based pediatric psychiatric data from Saudi Arabia remain scarce. This study assessed changes in psychiatric referral patterns and diagnostic trends before and after the pandemic in a major Saudi tertiary healthcare system and compared these findings with Gulf and international data.

## Methods

### Study design and setting

A retrospective cross-sectional study was conducted at Johns Hopkins Aramco Healthcare (JHAH), Dhahran, a tertiary healthcare system serving a multinational population.

### Participants

Children and adolescents aged 3–18 years referred for psychiatric assessment were included.

### Study periods

- Pre-COVID period: January–December 2019.
- Post-pandemic service-recovery period: January–November 2021.

### Exclusion of year 2020

During calendar year 2020, routine psychiatric outpatient clinics at JHAH were fully suspended due to COVID-19 emergency policy. Only emergency medical, surgical and obstetric services continued. Consequently, there were no routine psychiatric outpatient referrals during 2020.

Including 2020 would have artificially suppressed referral volumes artificially due to service closure not due to genuine reductions in psychiatric morbidity. Therefore, 2020 was intentionally excluded.

### Measures

Diagnoses were grouped using ICD-10 psychiatric categories.

### Classification of ‘other mental disorders’

In addition to the main ICD-10 diagnostic groups analyzed (depressive disorders, anxiety disorders, developmental disorders, behavioural disorders, sleep-wake disorders, feeding and eating disorders, and psychotic disorders), a further category labelled “Other mental disorders” was used to capture conditions that did not fit into the primary groupings above.

This category predominantly included:

- Somatoform and dissociative disorders (ICD-10 F44–F45).
- Stress-related and adjustment disorders (F43).
- Tic disorders and Tourette syndrome (F95).
- Elimination disorders such as enuresis and encopresis (F98.0–F98.1).
- Other childhood emotional and behavioural disorders not elsewhere classified (F93–F98), including.

- non-organic sleep disorders of childhood.
- selective mutism.
- non-specific behavioural/emotional symptoms.
- psychosomatic/functional presentations.

These diagnoses were applied by board-certified child and adolescent psychiatrists based on clinical assessment. This grouping was required because the individual numbers within each of these sub-diagnoses were too small to allow meaningful stand-alone analysis, but together they represented a clinically important subgroup of referrals.

### Statistical analysis

Descriptive statistics were calculated.

ANOVA with Bonferroni *post-hoc* testing and Kruskal–Wallis testing were applied where appropriate. p < 0.05 denoted significance.

## Results

### Demographic characteristics

Demographic and clinical characteristics are summarised in **Table 1**.

**Table 1:** Demographic and clinical characteristics of pediatric psychiatric referrals.

Variable	Frequency	Percentage (%)
Age (years)		
Mean ± SD	9.8 ± 4.5	—
Minimum–Maximum	1–17	—
Sex		
Male	1125	59.5%
Female	766	40.5%
Nationality		
Saudi Arabia	1428	75.5%
United States of America	130	6.9%
Canada	77	4.1%
United Kingdom	55	2.9%
Others	201	10.6%
Referral reason		
Developmental disorders	601	31.8%
Behavioural disorders	468	24.7%
Anxiety disorders	235	12.4%
Mood disorders	131	6.9%
Mixed anxiety & depressed mood	34	1.8%
Psychotic disorders	8	0.4%
Other mental disorders	112	5.9%
Non-mental-health diagnoses	129	6.8%
Routine exam without abnormal findings	173	9.1%

### Changes in referrals and diagnostic categories

Changes in referrals and diagnostic categories across study periods are presented in **Table 2**.

**Table 2:** Comparison of monthly referrals before and after the COVID-19 pandemic.

Diagnostic group	1st Half 2019Mean ± SD (Min–Max)	2nd Half 2019Mean ± SD (Min–Max)	1st Half 2021Mean ± SD (Min–Max)	2nd Half 2021Mean ± SD (Min–Max)	Test	p-value	Significant?
Developmental disorders	31.3 ± 9.1 (15–42)	17.2 ± 6.1 (10–26)	24.3 ± 7.4 (16–38)	27.3 ± 5.6 (22–34)	ANOVA	0.019	✓
Behavioural disorders	18.5 ± 5.5 (14–28)	13.8 ± 5.9 (6–21)	20.0 ± 7.3 (12–29)	25.6 ± 7.9 (15–37)	ANOVA	0.047	✓
Anxiety disorders	5.3 ± 1.8 (3–8)	4.7 ± 2.3 (2–7)	14.8 ± 7.1 (7–25)	14.3 ± 5.6 (4–19)	ANOVA	0.001	✓
Mood disorders	3.3 ± 2.1 (1–7)	2.6 ± 2.2 (0–6)	6.6 ± 3.9 (0–11)	9.2 ± 5.4 (4–18)	ANOVA	0.020	✓
Mixed anxiety & depression	Median 0 (0–1)	Median 1.5 (0–3)	Median 1.5 (0–3)	Median 2 (0–7)	Kruskal-Wallis	0.091	×
Psychotic disorders	Median 0 (0–1)	Median 0 (0–1)	—	Median 0.5 (0–2)	Kruskal-Wallis	0.272	×
Other mental disorders	2.3 ± 1.6 (1–5)	3.0 ± 1.7 (2–6)	4.6 ± 2.7 (2–8)	8.6 ± 2.9 (4–13)	ANOVA	0.001	✓
Total referrals	74.3 ± 16.7 (50–92)	52.3 ± 14.5 (30–69)	84.8 ± 20.9 (65–123)	103.6 ± 27.8 (57–135)	ANOVA	0.003	✓

1st half = Jan–Jun; 2nd half = Jul–Dec.

- Other mental disorders include psychiatric conditions that did not fall under the primary diagnostic groupings used in this study (mood, anxiety, developmental, behavioural, psychotic, sleep-wake or eating disorders). This category mainly comprised somatoform disorders, dissociative disorders, tic disorders, stress and trauma-related disorders, elimination disorders (enuresis/encopresis), and non-specific emotional or behavioural disorders coded in ICD-10 as F90–F98 and F40–F48 when not elsewhere classified.
- Statistically significant increases occurred across key categories and total referrals.
- Bonferroni *post-hoc* tests were used where applicable (already included in your original table text).

### Percentage change by disorder

Percent change in selected psychiatric diagnoses is shown in **Table 3**.

**Table 3:** Diagnostic percent change (2019 vs 2021).

Disorder category	ICD-10 codes	Percent increase (2021 vs 2019)
Depressive disorders	F30–F39	+91.6%
Sleep–wake disorders	F51, F99	+166.7%
Feeding & eating disorders	F50	+28.9%
Personality disorders	F60–F69	+162.5%

### Comparison with regional and global findings

A comparison with GCC and international findings is summarised in **Table 4**.

**Table 4:** Comparison with regional and global data.

Disorder category	Saudi Arabia (This Study)	GCC findings	International data
Depressive disorders	+91.6%	Increased post-lockdown symptoms in UAE (6)	~25% prevalence during pandemic (8)
Sleep–wake disorders	+166.7%	Sleep disruption reported in Qatar (7)	~34% pooled prevalence (12)
Feeding & eating disorders	+28.9%	Limited GCC pediatric data	Sharp rise globally (13, 14)
Personality disorders	+162.5%	Minimal GCC reporting	Rare reporting; likely under-identified

### Figures

**Figure 1:**
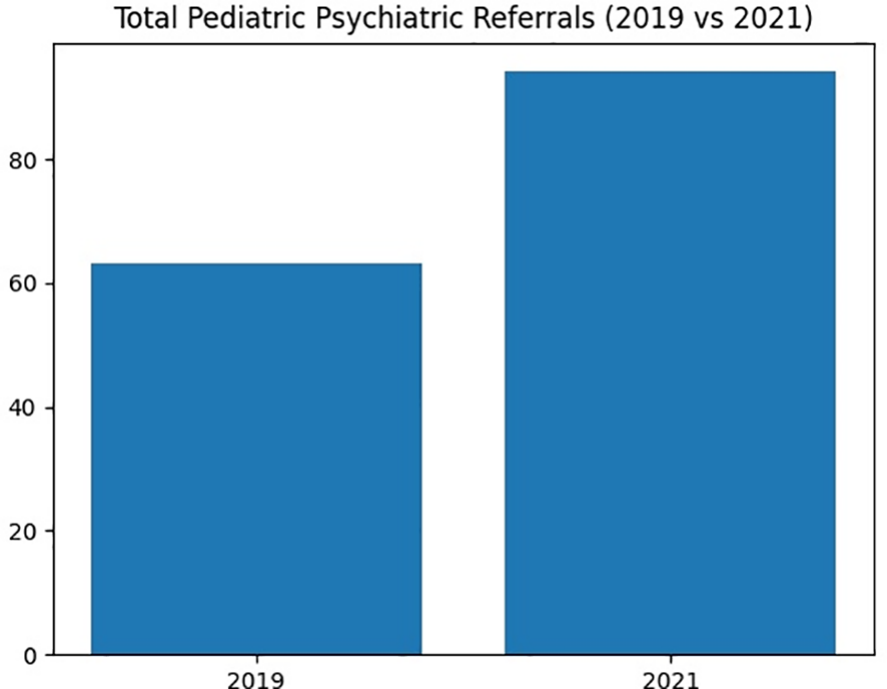
Total pediatric referrals (2019 vs 2021).

Total pediatric referral counts before and after the pandemic are illustrated in **Figure 1**.

**Figure 2:**
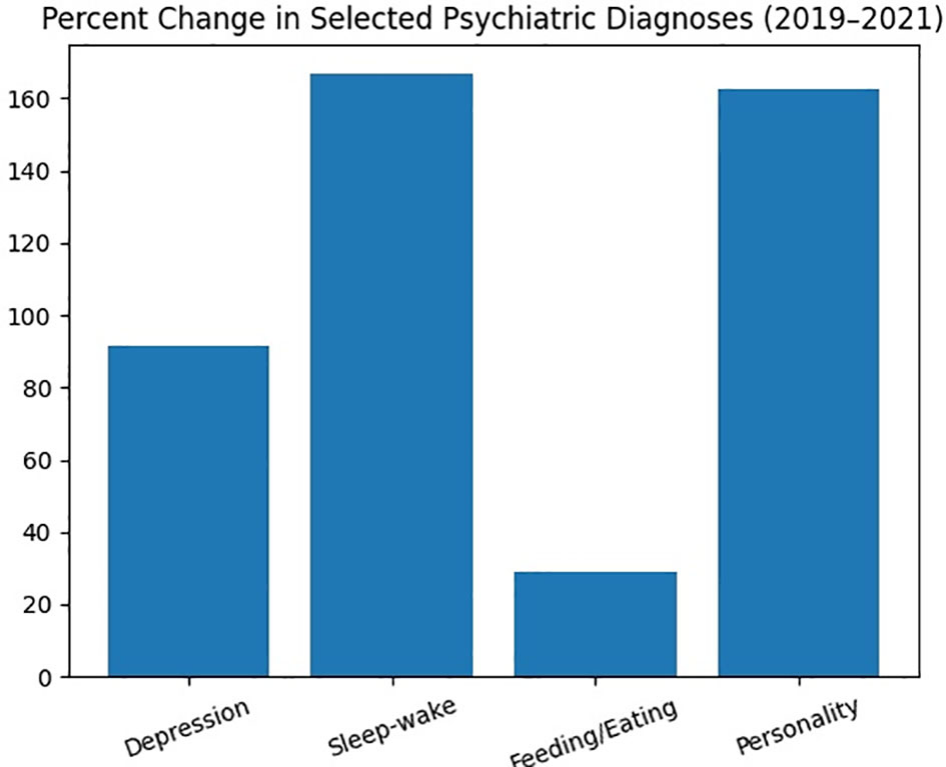
Percent change in selected psychiatric diagnoses (2019–2021).

Percent change across selected psychiatric diagnoses is shown in **Figure 2**.

**Figure 3:**
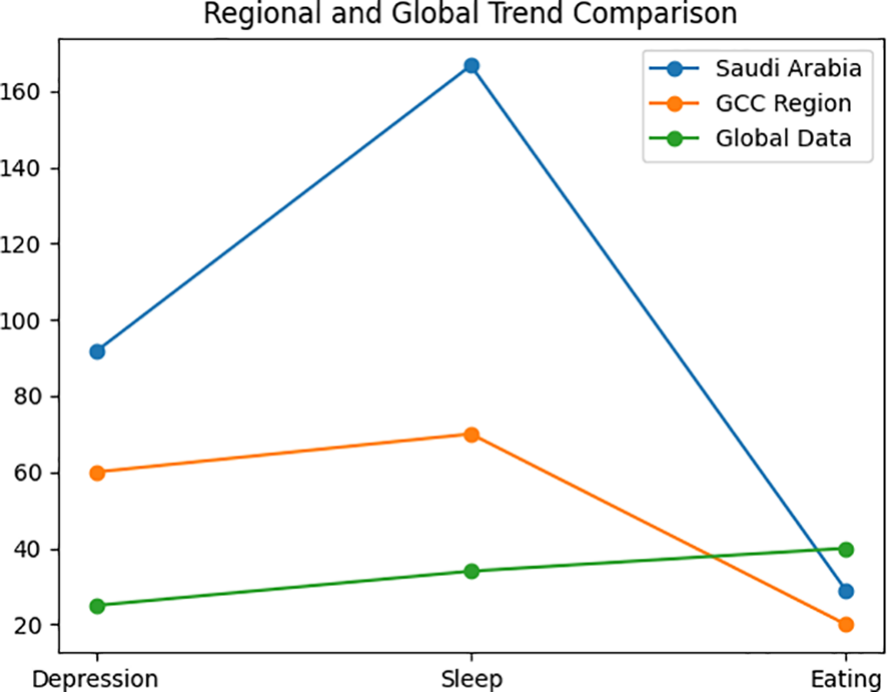
Regional and global trend comparison (with legend). (these data were obtained from heterogeneous sources and are intended for contextual illustration rather than direct quantitative comparison).

Regional and global trend comparisons are illustrated in **Figure 3**.

Figures are intended for descriptive visualization and do not represent inferential statistical comparisons.

## Discussion

This study demonstrated significant increases in total psychiatric referrals and in depressive, anxiety, sleep-wake, eating, and other mental disorders following the COVID-19 pandemic in a major Saudi healthcare system. These findings are consistent with regional Gulf studies reporting heightened psychological burden among children and adolescents following lockdown measures and school closures (5–7), as well as with international evidence documenting increased depressive and anxiety symptoms (8, 9), adolescent emergency-department crises (10, 11), sleep disturbances (12), and eating-disorder presentations (13, 14). These findings are also consistent with recent clinical evidence demonstrating increased psychiatric morbidity among young people following the pandemic (15).

### Interpretation

Although causal inference is limited by the retrospective nature of the design, the observed post-pandemic escalation in psychiatric referrals and diagnostic categories appears to be multifactorial. Prolonged social isolation, disruption of educational routines, and reduced peer interaction likely contributed to emotional dysregulation and depressive symptomatology (8, 9, 12). Increased reliance on digital media and screen exposure may have further exacerbated sleep-wake disturbances, attentional difficulties, and behavioural instability (12). In parallel, heightened family stress related to health concerns, financial uncertainty, and altered caregiving dynamics may have amplified vulnerability to anxiety and mood disorders (5–7, 9). Moreover, restricted access to mental-health services during lockdown periods may have delayed early identification and intervention, resulting in more severe or complex clinical presentations upon service resumption (10, 11). Collectively, these interconnected psychosocial and systemic stressors provide a coherent explanatory framework for the broad diagnostic escalation observed following the pandemic (15).

Personality-related diagnoses were observed predominantly among older adolescents and should be interpreted with caution. In this developmental context, such presentations may reflect stress-related behavioural dysregulation, emotional reactivity, or transient developmental responses rather than stable personality pathology (9, 10). The pandemic environment may therefore have unmasked vulnerability traits without necessarily indicating enduring personality disorders.

The category “Other mental disorders” represented a heterogeneous group including stress-related, functional, tic, elimination, and non-specific emotional and behavioural disorders. Although individually uncommon, the collective post-pandemic increase in these diagnoses suggests a broader functional and stress-related impact on child and adolescent wellbeing (5–7, 12). This pattern supports the concept that the pandemic influenced not only core psychiatric syndromes but also a wide spectrum of subthreshold and functional presentations.

### Limitations

Several limitations should be acknowledged. The single-center design limits generalizability, and the retrospective methodology restricts causal inference. The suspension of clinic services in 2020 required exclusion of that year, and referral practices as well as diagnostic behavior may have evolved following pandemic reopening. Nevertheless, exclusion of 2020 improved methodological validity by preventing artificial depression of referral rates and preserving temporal comparability.

### Clinical and policy implications

These findings underscore an urgent need to strengthen early identification and screening programs, expand child and adolescent mental-health service capacity, support family-focused intervention strategies, and prioritize vulnerable adolescents in post-pandemic recovery planning (5–7, 10). Investment in accessible, community-based, and school-linked mental-health services is essential to mitigate the long-term developmental consequences of pandemic-related psychological stress.

## Conclusion

The COVID-19 pandemic was associated with significant increases in psychiatric morbidity and referral demand among children and adolescents in Saudi Arabia. These data support investment in pediatric mental-health infrastructure and prevention strategies across the Kingdom.

## Data Availability

The raw data supporting the conclusions of this article will be made available by the authors, without undue reservation.
